# Functional Role of B Cells in Atherosclerosis

**DOI:** 10.3390/cells10020270

**Published:** 2021-01-29

**Authors:** Shelby D. Ma, Marion Mussbacher, Elena V. Galkina

**Affiliations:** 1 Department of Microbiology and Molecular Cell Biology, Eastern Virginia Medical School, Norfolk, VA 23507, USA; mas@evms.edu; 2 Institute of Pharmaceutical Sciences, Department of Pharmacology and Toxicology, University of Graz, A-8010 Graz, Austria

**Keywords:** atherosclerosis, B cells, adaptive immune system

## Abstract

Atherosclerosis is a lipid-driven inflammatory disease of blood vessels, and both innate and adaptive immune responses are involved in its development. The impact of B cells on atherosclerosis has been demonstrated in numerous studies and B cells have been found in close proximity to atherosclerotic plaques in humans and mice. B cells exert both atheroprotective and pro-atherogenic functions, which have been associated with their B cell subset attribution. While B1 cells and marginal zone B cells are considered to protect against atherosclerosis, follicular B cells and innate response activator B cells have been shown to promote atherosclerosis. In this review, we shed light on the role of B cells from a different, functional perspective and focus on the three major B cell functions: antibody production, antigen presentation/T cell interaction, and the release of cytokines. All of these functions have the potential to affect atherosclerosis by multiple ways and are dependent on the cellular milieu and the activation status of the B cell. Moreover, we discuss B cell receptor signaling and the mechanism of B cell activation under atherosclerosis-prone conditions. By summarizing current knowledge of B cells in and beyond atherosclerosis, we are pointing out open questions and enabling new perspectives.

## 1. Introduction

Cardiovascular diseases (CVDs) are the main cause of death worldwide. An estimated 17.9 million people died from CVDs in 2016 with the main cause for these deaths due to heart attack and stroke driven by developed atherosclerosis (World Health Organization report). Atherosclerosis is a disease of large- and medium-sized vessels that is characterized by the accumulation of cholesterol-rich lipoproteins, particularly low-density lipoprotein (LDL), within the inner walls of arteries, local inflammation, and formation of atherosclerotic plaques with areas of calcification and necrosis [1]. In 1856, Virchow initially suggested that the immune system might be involved in the pathogenesis of atherosclerosis [2]. Today, numerous evidence clearly indicates the importance of the innate and adaptive arms of the immune response in atherogenesis. Recently, clinical data from the CANTOS trial also demonstrate a cardiovascular benefit in high-risk patients who have been treated with canakinumab, a monoclonal antibody targeting IL-1β, highlighting the importance of inflammation in CVD development in humans [3].

Various immune cells are found within the aorta and surrounding adventitia at all stages of atherogenesis [4]. Quantitatively, the initial flow cytometric analysis of aortas demonstrated the presence of resident macrophages (MΦs) and vascular dendritic cells (vDCs) within the healthy vessels and these populations made up ~40% of total aortic leukocytes [5]. While functions of aortic resident MΦs remain formally not well investigated, it is likely that these cells play the role of a safeguard and are involved in the maintenance of tissue homeostasis and response to invading pathogens entering the vessel wall via the lumen or adventitial vasa vasorum [6]. Resident aortic MΦs might also utilize excess tissue cholesterol by upregulating desmosterol-induced LXR-dependent cholesterol efflux pathways, thus maintaining cholesterol homeostasis [7]. However, the overload of cellular cholesterol and a subsequent formation of cholesterol crystals result in pro-inflammatory MΦ expansion, foam cell formation, inflammasome activation, and impaired MΦ effector functions, all of which contribute to disease progression. Vascular DCs are found within the intima of the aorta and their distribution is shear stress-dependent [8]. Interestingly, intimal CD11c+ cells can serve as antigen-presenting cells and seem to be as effective as splenic DCs in antigen-dependent activation of T cells [9,10]. With the progression of the atherosclerotic plaque, further accumulation of modified LDL (mLDL) induces the recruitment of inflammatory monocytes that differentiate to either MΦs or vDCs serving as antigen-presenting cells that drive the T cell activation in atherosclerosis and further aggravate inflammatory responses [11]. T cells are mainly found in the adventitia of the healthy aorta, represent about 20% of total leukocytes in the aortic wall, and their relative numbers are increased in the atherosclerotic aorta [5]. The major phenotype of T cells in the aorta is IFNγ-producing T helper 1 (Th1) cells that play a pro-atherogenic role [12]. IL-17-producing CD4+ and IL-17+ γδ^+^T cells are also detected in the plaques, where they likely support the development of atherosclerosis via the regulation of myeloid cell migration to the aorta and provide plaque stability via TGFβ-dependent mechanisms; however, other studies also suggest a protective role of Th17 cells [13]. T regulatory cells (Tregs) are detected in the plaques as well and their atheroprotective role is firmly established [14], whereas the role of Th2 cells remains under debate [12]. It has been suggested that atherosclerosis-prone conditions may generate various antigens that can potentially activate T cells. Recent studies identified Apolipoprotein B (ApoB)—the core lipoprotein of LDL, very-LDL, and chylomicron—as an atherosclerosis-associated antigen that is recognized by CD4+ T cells in the context of proinflammatory cytokines and strong co-stimulation [15]. While it is likely that the main activation of adaptive immunity occurs in secondary lymphoid tissues, data suggest that local antigen presentation, activation of T cells, and induction of Tregs take place in the aorta and in tertiary lymphoid structures that are formed in the adventitia of aged atherosclerotic vessels [16]. The latest advantages in single cell RNA sequencing and mass cytometry opened new possibilities for identification and characterization of immune cells in the aortas. Recent studies from several groups revealed the complexity of the repertoire of aortic leukocytes and confirmed and extended data obtained by immunohistochemistry and FACS analyses of the aorta [17]. Overall, five types of MΦs, five types of T cells, two types of monocytes, three types of DCs, NK, ILC2 cells as well as B1 (CD79a and b, CCR7, and MZB1) and B2 (TPPP33, S100A6, and CD9) cells were found in the murine aorta [17].

## 2. B Cells in Atherosclerosis

### B Cells within the Aorta

Numerous lines of evidence demonstrate a vital role of B cells in atherosclerosis and highlight strong subset-specific effects of B1, Follicular (FO), and Marginal Zone (MZ) B cells, as well as regulatory B cells (Bregs) and innate response activator (IRA) B cells [18]. B cells are found within healthy and atherosclerotic aortas, surrounding the adventitia, and are present within tertiary lymphoid structures of the aortic wall [4,17]. CD19+ B cells represent about ~20% of the total number of aortic leukocytes and are located mainly in adventitia [5]. Under homeostatic conditions, B cells constitutively migrate to the aortic tissues via L-selectin-dependent mechanisms, and likely enter the adventitia via the vasa vasorum [5]. Inflammatory conditions further drive B cell recruitment to atherosclerotic plaques and initiate subsequent formation of arterial tertiary lymphoid tissues (ATLOs) at some areas of the vessel. In these structures, B cells consist of two populations: CD19+CD11b- B cells and a small population of CD19+CD11b+ cells [16]. Recent study demonstrates that CD19+CD11b+ B cells induce the impairment of T-cell antigen receptor (TCR) signal transduction and the promotion of TCR downregulation, inhibiting T cell responses [19]. Functions of this unique B cell population within ATLOs are not yet studied, but these CD19 + CD11b+ B cells might support a protective arm of these structures and suppress aortic T-cell dependent responses.

Various sets of chemokines and chemokine receptors direct migration of B cells to lymphoid and non-lymphoid tissues, such as CXCL13/CXCR5, CCL19/CCL21/CCR7, and CXCL12/CXCR4 pairs supporting B cell homing to sites of lymphoid structures [20]. CXCL13 and CCL21 are identified as chemokines that are crucial for the presence of B cells in ATLOs [21]. An additional role for CCR6 in the recruitment of B cells to the atherosclerosis-prone aorta that is controlled by inhibitor of differentiation-3 (Id3) has been recently revealed [22]. While several reports investigated mechanisms of B cell recruitment to the aorta, additional studies would need to focus on the identification of specific mechanisms for the recruitment of particular B cell subsets to the atherosclerotic aorta. For the successful development of B cell-associated therapies, we would need a much deeper understanding of how atherosclerosis-prone conditions affect chemokine receptors and the adhesion molecule repertoire of B cells, and particularly, B cell subsets.

## 3. BCR Signaling, B Cell Development, and Atherosclerosis

### 3.1. BCR Signaling

B cell development starts in the bone marrow by random rearrangement of the immunoglobulin heavy and light chains, creating a unique antigen-binding site of the B cell receptor (BCR) [23,24]. This event facilitates immature IgM+IgD+ B cells to leave the bone marrow, recirculate through secondary lymphoid tissues, and eventually become mature naïve B cells. The BCR consists of a membrane-bound immunoglobulin associated with a transmembrane heterodimer, Igα (CD79a) and Igβ (CD79b) [24,25]. B cell activation begins when the BCR binds to its specific antigen, which initiates phosphorylation of the immunoreceptor tyrosine-based activation motif (ITAM) by the tyrosine kinase Lyn. The phosphorylated Igα-Igβ recruit and activate Syk through their SH2 domain, thereby initiating the signaling cascade that eventually results in B cell activation [26]. Three main BCR downstream signaling pathways have been identified [26,27]: (i) the BTK pathway that is associated with B cell survival and differentiation through NF-κB activation, (ii) the mitogen-activated protein kinase/extracellular signal-regulated kinase (MAPK/ERK) pathway associated with proliferation through ERK activation, and (iii) the PI3K pathway associated with B cell differentiation, survival, and proliferation [28]. Furthermore, B cells can differentiate into antibody-secreting cells through extrafollicular differentiation, resulting in rapid plasma cell response or germinal center (GC) formation that is associated with high affinity antibody (Ab) production [26,29]. Although all details of BCR signaling are not fully understood, the ability to modify the BCR signal strength by directly targeting intrinsic BCR signaling molecules has provided insight into the role of BCR signaling in B cell differentiation and B cell functions [28].

### 3.2. BCR Signaling in Atherosclerosis

Despite the importance of BCR signaling in B cell activation and functions, very little is known about BCR-dependent B cell signaling and activation in the context of atherosclerosis. The presence of immunoglobulins against oxidation-specific epitopes (OSEs), development of GCs, and an increase in anti-oxLDL Ab-secreting plasma cells strongly support the idea that BCRs do recognize some OSE-related antigens in atherosclerosis [30,31]; therefore, they likely induce BCR-dependent signaling in the B cell. Several candidates have been discussed as potential self-antigens in the hyperlipidemic inflammatory environment of atherosclerosis. OSEs found on mLDL and apoptotic or necrotic cells can serve as some major antigens for BCR as they have been identified as a target of both IgG and IgM Abs. In addition, Ibrutinib, a BTK inhibitor used in cancer therapy, is clinically associated with increased risk of atrial fibrillation and hypertension, implying that the BCR signal can at least modulate atherosclerosis-associated risk factors [32].

### 3.3. BCR Signaling and B Cell Development

B cell development, survival, and differentiation into B cell subsets is dependent on constitutive signaling through the BCR [24,25,26] that consists of both antigen-dependent and antigen-independent (tonic) signaling. Tonic signaling is characterized by a low-level phosphorylation of signaling intermediates in resting B cells in the absence of robust and activating antigen triggers and this tonic signaling is driven by the surface expression of the BCR in B cells [33]. Both tonic and antigen-specific signaling are required for B cell survival [24,34,35]. To date, it is not known whether tonic BCR signaling is altered in atherosclerosis, and how tonic BCR signaling regulates B cell phenotypes in this disease; however, these are important questions that need to be answered.

The majority of newly generated B cells are autoreactive and despite numerous central tolerance mechanisms, i.e., receptor editing or clonal deletion, 30% of mature B cells remain autoreactive to some extent [36]. Indeed, it is now speculated that while highly autoreactive BCRs are removed from development, modestly autoreactive BCRs can escape the deletion [35]. In order to maintain peripheral B cell tolerance, activation of autoreactive B cells must be prevented: one mechanism for achieving this is the induction of B cell anergy, a state of unresponsiveness to self-antigen stimulation that is at least partially mediated by inhibitory BCR signaling [37]. IgM displays a higher sensitivity to endogenous antigens. Thus, the downregulation of IgM on maturing B cells might be a potential mechanism that supports the unresponsiveness of anergic B cells, whereas the expression of IgD still allows B cells to mount protective Ab responses against pathogens [38]. Currently, it is not known whether the anergic B cell phenotype is affected in atherosclerosis, and this essential question should be addressed in future studies. It would also be interesting to know how conditions of atherosclerosis that are enriched in endogenous antigens affect the distribution of surface IgM and IgD, and the subsequent sensitivity of B cells to BCR-induced signaling.

### 3.4. B Cell Subset Development and BCR Signaling

**Figure 1:**
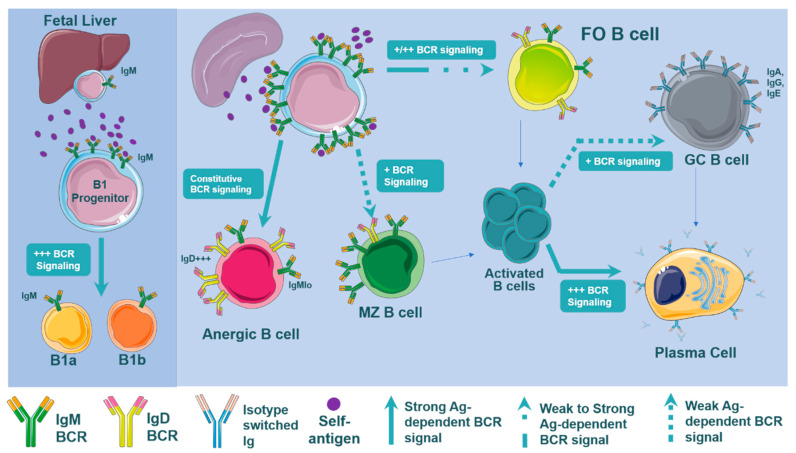
B cell development is dependent on B cell signal strength. Signaling through the B cell receptor (BCR) after (self)antigen recognition drives B cell differentiation through all stages of B cell development. Differentiation of B cells into B1, MZ, and FO B cells is mediated by positive and negative selection of BCR binding to antigens. B1 development is driven by strong BCR-derived signals. The spleen facilitates maturation of transitional IgM+IgD+ B cells to mature naïve MZ or FO B cells through negative selection: testing the autoreactiveness of the mature BCR. At this stage of maturation, highly reactive BCRs result in constitutive BCR signaling and fail to mature, instead, generating anergic B cells with an elevated surface IgD:IgM ratio. Transitional B cells that facilitate weak BCR signals preferentially differentiate to MZ B cells. In contrast, differentiation into FO B cells can occur with very weak or moderately strong BCR signals. The next stage of B cell development occurs after antigen binding and B cell activation. Weaker BCR signaling of activated B cells facilitates GC formation before becoming long lived plasma cells, while strong BCR signaling directs B cells to differentiate directly into short lived plasma cells. Surface expression levels of IgM and IgD and the types of antigen that are encountered mediate the activation of B cells and subsequent terminal differentiation.

To date, a lot of research has been performed to understand the origins and the biological functions of the different B cell subsets. Alone and in synergy with other receptors (e.g., BAFFR [39]) and signaling pathways (e.g., NOTCH-2 signaling [40]), BCR-derived signals drive the development of Follicular (FO), Marginal Zone (MZ), and B1 B cells (Figure 1). Several studies reported that enhanced BCR signaling, which is achieved via blockade of inhibitory pathways in the B cell, leads to an expansion of B1 populations [26]. Thus, B1 cell development requires a strong BCR stimulus and as a result, B1 cells are highly reactive to self-antigens [41,42,43]. It is also worth noting that enhanced tonic BCR signaling results in an increased B1 B-cell subpopulation and a dysregulated homeostasis of other B-cell subsets [44].

Preferential differentiation of immature transitional B cells into MZ or FO B cells in the spleen is still being uncovered (Figure 1). The general view is that the weaker BCR signaling results in the MZ B cell differentiation, while a stronger strength BCR signaling in transitional B cells results in the FO B cell development [35,45,46,47]. As a result, the development into the MZ B cell compartment tolerates a narrow range of modest self-reactivity, requiring self-reactive BCRs that will produce a signal strong enough to facilitate positive selection, but not so strong in order to stay under the negative selection threshold. In contrast, FO B cell development seems to tolerate a wider range of self-reactivity [35,48]. Either way, self-antigens that the BCR recognizes during B cell development play an essential role in shaping BCR repertoire and subsequent maturation. As such, we can speculate that the abundance of self-antigens presented in the atherosclerotic environment may likely affect BCR signaling, altering the B cell subset differentiation.

### 3.5. B Cell Subset Development in Atherosclerosis

**Figure 2:**
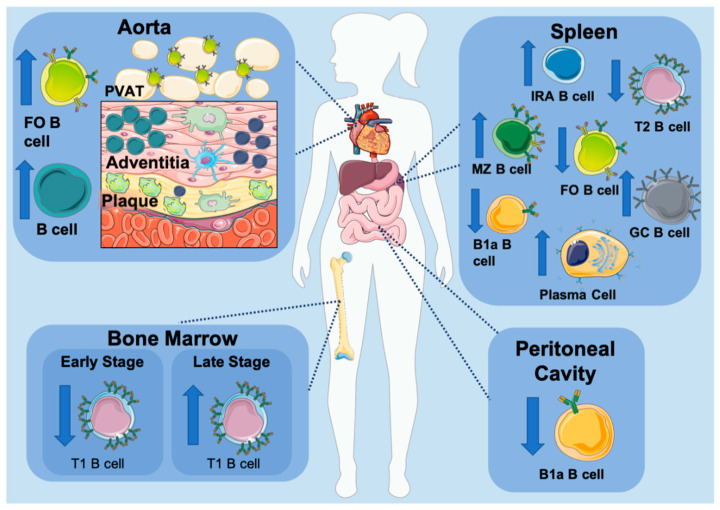
B cell subset development in atherosclerosis. Several animal studies have demonstrated that an atherosclerotic environment modulates B cell subset composition, evident by the altered distribution of B cells throughout the body. Artery tertiary lymphoid tissue (ATLO) located directly adjacent to plaques and the perivascular adipose tissue (PVAT) that surrounds the aorta contain an elevated number of B cells. The number of transitional B cells (T1) in bone marrow correlates with the progression of atherosclerosis with a decrease in early stage and increase in late stage. B1a B cells residing in the peritoneal cavity are decreased, while IRA B cells are significantly elevated in atherosclerosis. The spleen is the main reservoir of B cells and a center of humoral responses. Despite the decrease in transitional B cells (T2), MZ B cells are significantly increased in atherosclerosis. FO B cells are shown to either decrease or have no significant change despite the significant increase in germinal center (GC) B cells and plasma cells.

What is the role of BCR signaling in B cell differentiation in atherosclerosis? So far, there is no direct evidence demonstrating that BCR signaling affects the splenic B cell subset composition in atherosclerosis. However, several studies have shown that the development of B cells is either directly or indirectly modulated by atherosclerosis (Figure 2). This is evident by an increase in splenic transitional B cells, MZ B cells, and a decrease in FO B cells in atherosclerosis [30,31,49]. Some recent reports indicate the implication of NKT cells, the CD23 receptor, and BCR regulatory molecules in B cell subset differentiation in atherosclerosis. Soh and colleagues reported that hypercholesterolemia induces an increase in MZ B cells via the prevention of apoptotic MZ B cell death due to attenuated expression of IFNγ by NKT cells [30]. Interestingly, when investigating sialic acid-binding immunoglobulin-like lectin G (Siglec-G) as a potential target for chronic inflammation, Gruber et al. discovered that B cell-specific deficiency of Siglec-G results in reduced atherogenesis due to the expansion of B1a cells in the peritoneum [50]. Siglec-G is known as a negative regulator of BCR signaling in B1 cells [51]; therefore, it is quite possible that the loss of Siglec-G enhances the B1 cell population, suggesting that a strong BCR signaling can induce an atheroprotective B1 cell phenotype. Recently, it has been shown that secreted IgM regulates B cell development by acting as a decoy for self-antigen [47]. It also seems that surface expression of IgM plays an important role in the direction of B cell subset differentiation [38,52]; mice lacking secreted IgM displaying increased BCR signaling developed increased MZ and decreased FO B cell numbers [47,53]. In atherosclerosis, loss of secreted IgM results in accelerated lesion formation due to reduced levels of B cells expressing the low-affinity IgE receptor CD23 and elevated IgE synthesis [54].

A recent study by Nus and colleagues further highlighted a potential role for a BCR-dependent B cell differentiation and activation in atherosclerosis [55]. Nur77 (nerve growth factor IB) is a highly induced transcription factor upon BCR engagement in B cells, reflecting the levels of BCR-dependent B cell activation. Interestingly, MZ B cells express high levels of Nur77 in atherosclerosis, suggesting that the atherosclerotic environment provides self-antigens that are sensed by MZ B cells, leading to their constant stimulation and active involvement in immune response. A study by Nus and colleagues supports this notion, demonstrating the protective role of MZ B cells in atherosclerosis is at least partially due to upregulation of programmed cell death ligand-1 (PDL-1) (discussed in B cell subsets in atherosclerosis), which is in turn controlled by relatively strong BCR signaling [56,57]. In contrast, Nur77 was significantly decreased in FO B cells, implying that FO B cells are encountering less self-antigens in atherosclerosis or that B cell activation is regulated differentially in this B cell subset. In line with that, Nus et al. found that FO B cells upregulated ICOSL, a major inducer of Tfh-cell differentiation in atherosclerosis [55], which is facilitated by lower BCR signal strength [57]. Differentiation into short-lived plasma cells (SLPC) or GC B cells is also determined by a BCR signal strength [58,59,60,61]. Strong signaling through the BCR drives SLPC expansion, while weaker BCR signaling facilitates GC formation. GC B cells along with Ab-secreting cells are increased in an atherosclerotic environment. Importantly, MZ B cells, upon synergistic stimulation of Toll-like receptors (TLR) and BCR, can facilitate GC formation instead of differentiating into SLPC [62]. Together, initial studies suggest an involvement of BCR signaling in modulation of B cell subset differentiation in atherosclerosis (Figure 2). Thus, future studies should focus on better understanding of how BCR signaling impacts signals downstream of the BCR, shapes B cell differentiation and functions, and what are cooperative roles of other signaling events, such as chemokines or co-receptors, TLRs, and microbiota-associated signals in the differentiation and maintenance of B cell subsets in atherosclerosis.

### 3.6. Toll Like Receptors, BCR Signaling, and Atherosclerosis

Additional difficulties in understanding the BCR’s role in atherosclerosis is the involvement of TLRs in the B cell activation and differentiation. TLRs are important innate immune receptors that recognize pathogen-associated molecular patterns (PAMPs) and damage-associated molecular patterns (DAMPs), such as OSEs. B cells are unique in that they express both an antigen-specific BCR and a germline-encoded TLR, where dual engagement allows them to be a part of the innate and adaptive immune response [63]. Although TLRs are shown to be irrelevant for B cell development, it is clear that the addition of TLR engagement to BCR stimulation can influence the maturation of B cells and their functional responses [63]. TLR stimulation on B cells can overcome the BCR-stimulated negative selection-induced apoptosis of immature/transitional B cells, instead improving B cell proliferation, survival, and differentiation, rescuing autoreactive cells [64,65]. Functionally, TLR stimulation, specifically signaling via MyD88, can influence terminal differentiation into SLPC or class switching and affinity maturation, upregulate co-stimulatory molecules important for T cell help, induce cytokine production and MHC-II expression required for B cell antigen presentation, and upregulate integrin expression important for B cell migration [62,66,67]. Not only are these functional responses specific to the stage of B cell developmental, but also are B cell subset-specific [65]. While BCR stimulation alone can induce preferential differentiation of B1 and MZ B cells into IgA- or IgM-producing plasma cells, respectively, dual engagement of the BCR and TLR leads to a much more rapid induction of these protective Ab secreting cells [63]. Additionally, TLR stimulation can induce MZ B cell migration into the follicles, B1 B cell migration out of the peritoneal cavity, and support MyD88-dependent [68,69] class switch recombination, GC proliferation, and earlier Ab production [63,70]. Thus, the integration of dual TLR and BCR signaling clearly influences B cell responses.

In CAD, signaling through TLRs is shown to contribute to the pathology through activation of MAPKs and NF-κB pathways, resulting in the upregulation of proinflammatory cytokines and chemokines, and the initiation of inflammatory responses [71,72,73]. Studies on TLR deficiency have shown that the effect on atherosclerosis development depends on the type of TLR [73,74,75,76]. TLR9 appears to play a protective role in the progression of atherosclerosis [77], while TLR4 can either protect or accelerate plaque burden [75,76]. Surprisingly, only a few studies have specifically investigated the role of TLRs in B cells in atherosclerosis. Hosseini et al. demonstrated that the atheroprotective production of IgM by peritoneal B1a B cells is dependent on TLR4/MyD88 expression [78]. Another study by Karper et al. investigated RP105, a TLR regulator, in atherosclerosis and found that RP105 deficiency resulted in decreased lesion formation in parallel to altered B2 B cell activation, indicating an indirect role of TLR in B cells [79].

Although it is evident that both TLR and BCR stimulation affects atheroprogression, it is still unclear what the exact antigens are that stimulate these receptors in atherosclerosis. In general, TLRs have a wide range of exogenous and endogenous ligands that can stimulate the signaling cascade such as LPS, HSPs, and oxLDL, all of which are upregulated in atherosclerosis. The responsiveness of B cells to TLRs can be modulated by BCR signaling molecules, while control of B cell autoreactivity can be affected by TLR engagement [80]. The inflammatory environment in atherosclerosis likely leads to increased synergistic effects of BCR and TLR engagements that can change B cell development and functions and subsequently affect atherogenesis. The challenge ahead lies in the identification of the BCR signaling events and characterization of B cell-specific TLR ligands in atherosclerosis with an explicit focus on B cell subsets that potentially might have a unique combinatory response to BCR and TLR engagements.

## 4. B Cell Subsets in Atherosclerosis

B cells are a heterogeneous group of cells that originate either from the bone marrow or arise during embryonic development (Figure 1). B1 B cells, which can be phenotypically and functionally divided into B1a and B1b cells, predominately originate from progenitors found in the fetal liver, while B2 B cells originate from bone marrow progenitors [25,81]. These B2 B cells are further differentiated into FO and MZ B cells. Other less investigated B cell subsets include IRA B cells and Bregs. In the context of atherosclerosis, it is well established that the role of B cells is subset-specific with IRA and FO B cells promoting atherogenesis, while B1 and MZ B cells protect against atherogenesis [18,82].

B1 cells are self-renewing B cells that are primarily localized in the peritoneal and pleural cavities where they significantly contribute to the plasma’s natural IgM levels [42,81]. There are two subsets of B1 B cells based on the expression CD5 expression: CD5+ B1a cells and CD5- B1b cells (Table 1). B1a B cells spontaneously produce IgM in T cell-independent (TI) responses, whereas B1b cells can be involved in both TI and T cell-dependent responses, and T cell-dependent responses provide long-lasting IgM memory to various pathogens [83]. Interestingly, B1b cells can also show IgA isotype switch and have relatively high frequencies of somatic mutations in IgA-associated heavy-chain variable regions [84]. B1a and B1b B cells are atheroprotective mainly due to the production of IgM Abs that can bind to OSE in LDLs, apoptotic cells, or cell wall polysaccharides of pathogens, such as *Streptococcus pneumonia* [85,86]. Studies showed that a reduction in B1a cells aggravates atherosclerosis, while adoptive transfer of B1a or B1b cells reduces atherogenesis [87,88]. Mechanistically, B1 IgM-expressing B cells reduce uptake of oxLDL by MΦs and stabilize atherosclerotic plaques by increasing the number of TGFβ1-expressing MΦs, which clear apoptotic cells and shift the balance towards reduced TNFα, IL-1β, and IL-18 levels.

IRA B cells are derived from B1a cells and develop in the spleen in response to LPS stimulation. They are characterized by their secretion of granulocyte macrophage colony stimulating factor (GM-CSF) [91], which is responsible for their proatherogenic effects in atherosclerosis [92]. Hildengorf et al. found that mixed chimeric mice lacking B cell-derived GM-CSF (IRA B cell-deficient) develop smaller atherosclerotic lesions containing fewer MΦs and effector T cells [92]. Moreover, IRA B cell-derived GM-CSF increases DC activation and aggravates atherosclerosis by shifting adaptive immune responses toward a more pro-inflammatory atherogenic phenotype.

Initial studies on the role of B2 B cells (FO and MZ B cells) in atherosclerosis showed a protective role of B cells. Splenectomy dramatically aggravated atherosclerosis in *Apoe*^−/−^ mice and adoptive transfer of splenic B cells reduced plaque burden in splenectomized *Apoe*^−/−^ recipients [90]. B cell deficiency was associated with an increased plaque burden in *Ldlr*^−/−^ mice [98]. Doran and colleagues found that adoptive transfer of B2 B cells into B cell-deficient atherosclerotic mice led to a decrease in atherosclerosis [22]. In contrast, depletion of B2 cells was associated with reduced progression of atherosclerosis caused by a decrease in proatherogenic Th1 cells in spleen and atherosclerotic plaques [95,99]. In line with this evidence, Kyaw et al. found that reintroduction of B2 cells into B cell-deficient mice leads to aggravated atherosclerosis [96]. In addition, production of high affinity IgG and IgE have been shown to aggravate atherosclerosis by promoting macrophage and mast cell inflammatory response in atherosclerotic lesions [18]. The discrepancies between these studies can be attributed to multiple factors: Doran et al. adoptively transferred B2 B cells isolated from atherosclerotic prone *Apoe*^−/−^ mice, while Sage et al. isolated B2 B cells from healthy C57BL/6 mice. As it is now established that the relative proportion between FO and MZ B cells is altered upon atherosclerosis development, it is possible that B2 cells from C57BL/6 vs. *Apoe*^−/−^ donor mice had different proportions of proatherogenic FO and atheroprotective MZ B cells. Additionally, B2 B cells isolated from *Apoe*^−/−^ mice were exposed to the inflammatory and lipid-rich environment prior to injection and these conditions could affect B cell functions and reactivity.

As B2 cells consist of FO and MZ B cells, further studies demonstrated specific roles for B2 B cell subsets in atherogenesis. FO B cells, which predominately reside in the follicles of the spleen, make up the majority of resident splenic B cells and form the main population of mature B2 cells [45]. FO B cells support the Th1 response and its production of proinflammatory cytokines [100]. In addition, activated by Tfh cells, FO B cells differentiate into GC B cells that are involved in GC formation [25]. Production of high affinity antigen-specific IgG and IgE is the result of GC B cell proliferation and affinity maturation [101]. A number of studies have demonstrated a proatherogenic role of FO B cells mainly via the production of IgG and the activation of Th1 cells [100]. To date, it is not completely understood how much of the resulting phenotype of FO B cells results from the production of IgGs, direct effects on T cells via an antigen presentation to support T effector responses, or its impact on other antigen-presenting cells.

MZ B cells are the smallest subset of B2 B cells and their specialized location in the outer white pulp of the spleen allows them to respond rapidly to bloodborne pathogens and other T-cell-independent antigens, typically through TLR signaling [45,102]. So far, accumulating data indicate that MZ B cells play a protective role in atherosclerosis likely via two different pathways: suppression of Tfh cells and production of protective IgM Abs [55]. Mechanistically, atherosclerotic conditions upregulate the cAMP-dependent transcription factor ATF3, which induces the surface expression of PDL-1 on MZ B cells, thereby suppressing Tfh cell motility, Tfh differentiation and abundance, and proatherogenic Tfh cell-mediated responses [56]. The importance of MZ B cells as IgM-secreting cells is less understood; thus, insights into the contribution of MZ B cell-derived IgM Abs would be critical for the understanding of the atheroprotective mechanism of MZ B cells.

## 5. B Cell Functions in Atherosclerosis

### 5.1. Role of Immunoglobulins in Atherogenesis

**Figure 3:**
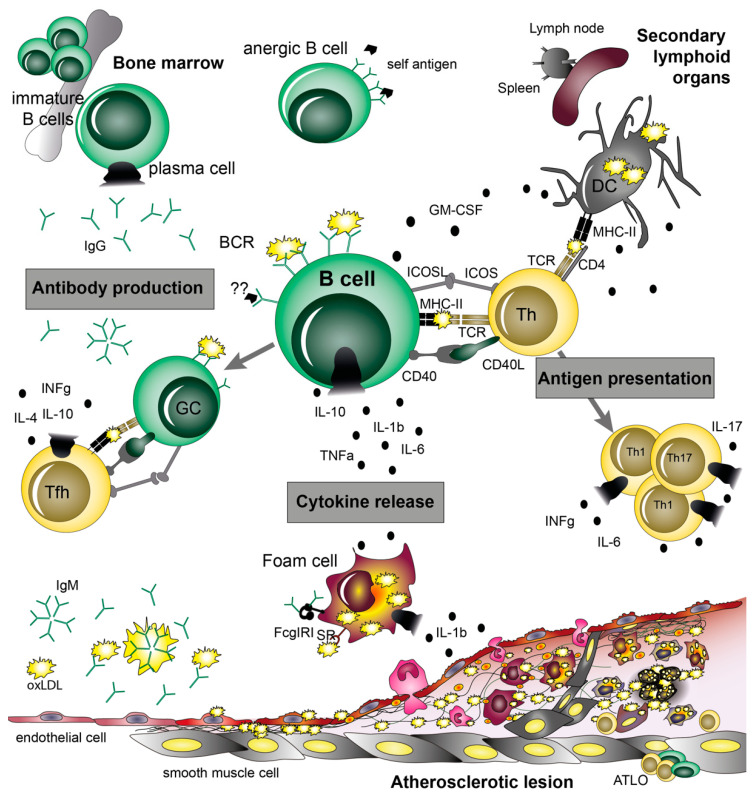
B cell functions in atherosclerosis. B cells can play both atheroprotective and pro-atherogenic roles, which are mediated by their main effector functions: (i) antibody production, (ii) cytokine release, and (iii) antigen presentation/interaction with T cells, which are dependent on their subset and activation state. By detecting (self)antigens via their specific B cell receptors (BCRs), B cells become activated and differentiate into antibody-producing plasma cells. This process is supported by the interaction of germinal center (GC) B cells and T follicular helper (Tfh) cells and results in the release of immunoglobulins (Ig). Ig can activate various cell types (e.g., macrophages) by binding to their respective Fc receptors (FcR), neutralize oxidized lipoproteins (oxLDL), and initiate complement activation and opsonization. Moreover, via release of cytokines such as TNFα, IL-6, IL-1β, GM-CFS, and IL-10, B cells are able to affect their microenvironment in a pro- and anti-inflammatory way. In secondary lymphoid organs (spleen and lymph nodes) and arterial tertiary lymphoid tissues (ATLOs), interactions between B cells and Th cells take place via binding of co-stimulatory molecules (e.g., ICOS/ICOSL, CD40-CD40L) as well as antigen presentation of B cells via MHC-II.

B cells shape the immune response through three main functions: antigen presentation, cytokine production, and immunoglobulin synthesis, and thereby participate in systemic and local immune responses in atherosclerotic arteries (Figure 3). One of the important functions of B cells is to secrete antigen-specific immunoglobulins and provide a balanced immune response in homeostasis and under pathological conditions [101]. B cells produce five classes of immunoglobulins—IgM, IgD, IgG, IgA, and IgE—that are distinguished according to the C-terminus regions of their heavy chains, which are constant and therefore, do not participate in antigen binding but serve for the effector functions of Abs. IgG Abs also display four subclasses or isotypes of IgG Abs—IgG1, IgG2, IgG3, and IgG4 in humans and IgG1, IgG2a, IgG2b, and IgG3 in mice [101]. The major effector functions of Abs include: neutralization of a target, opsonization of antigens, and activation of MΦs and other cells via binding to Fc receptors (FcR), or initiating the classic pathway of the complement system by binding to C1q. Heavy chain isotype and binding affinities of activating and inhibitory FcR on immune cells would determine the dominance of the effector function [101].

### 5.2. IgM

IgMs are the first Abs that B cells express during their development [103]. The first location of IgM expression is bone marrow where heavy and light chains on naïve B cells form a cell surface IgM that serves as a part of the BCR complex [34]. Membrane-bound IgM participates in the processes of antigen-recognition, induction of B cell activation cascades, and homeostatic fitness of B cells via activation of PI3K [104,105,106]. Secreted IgM circulates in two different forms as natural (non-specific) IgM and antigen-specific IgM [107]. Peritoneal B1 cells produce a large amount of natural IgMs, which are polyreactive Abs that bind self-antigens such as phospholipids as well as foreign antigens (antigens on *Streptococcus pneumonia*, *Influenza*, *Borrelia hermsii*, and *Salmonella*, among others) [108]. Moreover, IgMs are well recognized for their ability to bind to various cell surface receptors including complement receptor CR2 and CR3, polymeric Ig receptor (pIgR), Fcα/μR and FcμR on B-cells, epithelium cells, and antigen-presenting cells, supporting a fine-tune of B cell-mediated responses [109].

Like B1 cells, MZ B-cells are also considered as innate-like cells that play an important role in homeostasis, supporting the clearance of apoptotic cells and cellular debris via production of a limited Ab repertoire, mainly recognizing common bacterial motifs and self-antigens. MZ B-cells can produce natural Abs and Abs in response to T cell-dependent and T cell-independent antigens [101]. After interacting with antigens, MZ B cells rapidly differentiate into plasmablasts that produce large amounts of low-affinity IgM [110]. The formation of IgM-expressing plasmablasts is facilitated by an elevated baseline expression of BLIMP-1 [110]. Recently, long-term plasma IgM-producing cells with a protective phenotype against lethal virus have been found within the spleen [111]. These cells are somatically mutated, likely antigen-induced, and are distinct from either short-lived IgM+ plasma cells or natural IgM+ B1 cells. While quite a lot of work has now been done on the role of IgM and B1a and B1b cells in atherosclerosis, the specific role of IgM+ plasma cells in atherosclerosis is not well understood and future studies that would uncover the fate of IgM-producing plasma B cells in this disease are necessary and important.

### 5.3. IgM in Atherosclerosis

One of the most prominent and well-characterized antigens in atherosclerosis are oxLDLs [112]. oxLDLs contain OSEs that represent a variety of epitopes in oxidatively modified self-proteins and lipids. OSEs, including oxidized phospholipids (OxPLs) and malondialdehyde (MDA)-modified amino groups, have also been documented on the surface of apoptotic cells and microvesicles [113,114]. The current working concept in the field is that the immune system senses and clears these structures in homeostasis, but overwhelming conditions of induced inflammation break the protective response and then increasing amounts of generated OSEs initiate proatherogenic immunity.

One of the first indications that the immune system has developed a protective mechanism against potentially dangerous OSEs came from a study by Palinski and colleagues, demonstrating the existence of circulating natural Abs against oxLDL in *Apoe*^−/−^ mice [115]. Generated from *Apoe*^−/−^ mice, B-cell clones produced IgM Abs to oxLDL, termed E06 Abs. These Abs recognize oxidized phospholipids containing the phosphorylcholine headgroup in oxLDL and apoptotic cells [85,112,116]. Importantly, E06 Abs are functionally active as they block oxLDL uptake by MΦs [117]. Transgenic overexpression of the single chain variable fragment of E06 results in robust and preferential increase in OSE-specific IgM Abs and attenuated atherosclerosis in *Ldlr*^−/−^ mice [118]. Interestingly, passive transfer of polyclonal IgM also reduces atherosclerosis in *Apoe*^−/−^ mice [119].

The antigen binding site of E06 Abs is the same as for natural T15 Abs, which are specific for phosphorylcholine and protect from virulent pneumococcal infection [85]. This evidence suggests the existence of a naturally occurring specific B1 cell pool that is responsible for both OSE-specific Abs and represents a mechanism of a protective clearance of damaged cells, pathogens, and mLDL in heath and diseases. In line with this idea, the functional importance of T15 IgM Abs and its cross-reactivity with OSE epitopes of oxLDL and bacterial infections was demonstrated in elegant studies by Binder and colleagues [120]. The immunization of *Ldlr*^−/−^ mice with *Streptococcus pneumonia* led to elevated levels of oxLDL-specific IgM and an expansion of oxLDL-specific splenic T15 IgM-producing B cells, which were cross-reactive with pneumococcal determinants, and subsequent weakened atherogenesis [120]. Studies with the *Ldlr*^−/−^ mice deficient in soluble IgM (sIgM) further highlighted the overall protective role of sIgM as these mice developed substantially larger and more complex lesions with profound cholesterol crystal formation and increased smooth muscle cell content in aortic root lesions. The protective effects of sIgM seem to be independent of the classical pathway of complement activation and rate of effective efferocytosis [121]. Interestingly, sIgM deficiency resulted in increased levels of plasma IgE, activation of mast cells, and accelerated atherosclerosis development [121].

### 5.4. B1a and B1b Cells Produce OSE-Specific IgM

E06 and T15 are found within advanced lesions of atherosclerosis-prone mice [85,86]. B1 cells including both B1a and B1b cells can produce specific IgM Abs against OSEs. There are several anatomical locations of B1 cells including bone marrow, peritoneal cavity, and spleen, in which the synthesis of OSE-specific IgM Abs is detected. Although mechanistic characterization of signals driving a specific production of OSE-specific IgM Abs by B1 cells is in its infancy, it is becoming clear that several factors regulating B1 activities exist. While the production of T15 Abs against *Streptococcus pneumonia* is dependent on the V1 (VHS107.1.42) immunoglobulin heavy chain gene, IgM/T15-dependent protection against atherosclerosis is independent of the use of the VHS107.1.42 gene [122]. Another feature of E06 Ab secretion is its dependency on CD1d, but not via iNKT-dependent pathways [123]. The E06 but not total IgM Abs are selectively increased in *Cd1d*^−/−^ mice due to the expansion of splenic E06-producing B cells. The existence of a subpopulation of short-term IgM-producing plasma cells within the mantle zone and a pool of long-term IgM+ plasma cells is becoming appreciated. It is possible that these subsets are selected for their propensity to elicit IgM-protective mechanisms against OSE-containing antigens. Clearly, more studies are needed to uncover a mechanism of differentiation and functions of splenic short and long-term plasma cells producing IgM in atherosclerosis.

While several studies identified the importance of B1 cells in the spleen, additional reports also revealed the importance of B1 cells for IgM synthesis in the bone marrow. Two studies have identified CXCR4 as a critical regulator of B1 cell migration to the bone marrow, as CXCR4 deficiency resulted in decreased B1 cells in the bone marrow, reduced IgM levels [124], and elevated atherosclerosis in B-cell-specific CXCR4-deficient female *Apoe*^−/−^ mice [125]. In line with these observations, CXCR4 expression on B1 cells positively correlates with MDA-modified LDL-specific IgM Abs and adversely with coronary plaque burden in humans [124].

The functional role of anti-oxLDL-specific IgM Abs and thus a protective role of B1 cells including B1a and B1b has been revealed in several studies using atherosclerosis-prone mice. While both B1a and B1b cells produce IgM Abs and are developed in processes of positive selection against self-antigens, little is known about potential differences between these two subsets. Recently, Prohaska and colleagues utilized an unbiased 5′ RACE amplification strategy with massively parallel sequencing to define the IGHV functional repertoire of all murine peritoneal and splenic B cells [126]. This study revealed that B1a cell sequences differed from the B1b subset, suggesting a distinct selection process for both these subsets in C57BL/6 mice. It is not clear yet how the repertoire of B1a and B1b subsets is impacted by inflammatory hyperlipidemic conditions in atherosclerosis and which signals might be involved in the induction and control of IgM production by B1a vs. B1b cells in this disease.

Taking into account the prominent role of natural IgM and specifically E06/T15 Abs in the regulation of atherogenesis, several independent studies demonstrated the importance of B1a cells and natural IgM in atherosclerosis. Kyaw and colleagues showed that splenectomy-induced depletion of peritoneal B1a cells augmented atherosclerosis but interestingly, has no impact on lesion composition or plaque stability [87]. Adoptive transfer of B1a, but not B2 cells, restored protective responses and decreased rates of plaque burden in the splenectomized mice to the levels of sham-operated mice [87]. These protective effects of B1a cells were due to the secretion of IgM as B1a cells with only surface, but not soluble, expression of IgM [127] had no effects on plaque development. Siglec-G was a focus of recent studies as it negatively regulates the B1a cell content likely via downregulating BCR-dependent signaling. Siglec-G deficiency supported B1 cell development and production of OSE-specific IgM Ab [50]. IL-5 is an essential cytokine for B1 cell expansion and IgM production [128]. Importantly, B1 cells require IL-5 for proper expansion and release of oxLDL-specific IgM in atherosclerosis [129].

In contrast to B1a cells, B-1b cells can be activated by both non-antigenic and antigen-dependent stimuli, producing some antigen-specific IgM and IgG3 [130]. Adoptive transfer of B1b B cells into *Rag-1*^−/−^*Apoe*^−/−^ recipients resulted in the attenuated atherosclerosis and increased the production of OSE-specific IgM Abs [88]. Notably, TLR4-activated B1b cells can also produce OSE-targeted IgM [88]. Together, these data suggest a multifactorial mechanism, which is involved in the fine-tune regulation of selection, maintenance, and functions of B1a and B1b cells in atherosclerosis. While current reports clearly indicate that B1a and B1b cells serve as the atheroprotective populations via their production of sIgM, identification of BCR-dependent signals, particularly the effects of tonic signaling, and the biological context that could lead to B1 cell expansion and activation in atherosclerosis, should be an important future research goal for the field.

### 5.5. IgE and Atherosclerosis

IgE was the last discovered Ig class [131]. Despite small amounts of circulating IgE in body fluids and limited numbers of IgE-positive cells, IgE is a powerful mediator of allergic disease and host defense. In general, IgEs are developed against helminths and support a predominant Th2 response, as well as mast cell and eosinophil activation [132]. Therefore, IgEs are powerful inducers of multiple deleterious effects in allergies and autoimmunity [133]. The production of IgE is positively regulated by IL-4, IL-13, and IL-9, and tightly controlled by Tregs, IFNγ TGFβ, and by several intrinsic mechanisms providing short-lived IgE plasma cell differentiation.

It is quite surprising, but data indicate that elevated levels of IgE serve as an independent predictor for coronary heart disease. Deposition of IgE and FcεR1 are increased in human and mouse atherosclerotic plaques, suggesting a direct role of IgE in the regulation of inflammation within atherosclerotic lesions [134]. IgE binds to FcεR1 and FcεR2 (CD23) expressed on mast cells, basophils, and other leukocytes and can induce strong activation of the target cell. As FcεR1 and TLR4 interactions induce MΦ activation, FcεR1 deficiency attenuates MΦ signal transduction, inflammatory gene expression, apoptosis, and diminishes atherosclerosis [134]. Follow up studies directly tested a role of IgE in vascular cells using IgE-deficient (*Ige*^−/−^) *Apoe*^−/−^ mice [135]. *Ige*^−/−^ *Apoe*^−/−^ mice demonstrated attenuated plaque burden, which was accompanied by a reduction in M1 MΦs, smooth muscle cells, and microvessels [135]. Intriguingly, IgE also reduced MΦ-sterol-responsive network gene expression and promoted MΦ cholesteryl ester intracellular accumulation and foam cell formation and supported the development of obesity and diabetes mellitus, linking the development of atherosclerosis to metabolic diseases [135].

### 5.6. IgG

IgG is one of the most abundant proteins in human serum (~10–20% of plasma protein) and is the major class of immunoglobulins. Overall, there are four IgG subclasses with different structures and functions, with the most abundant being IgG1 [136]. Generation of IgG subclasses is antigen-dependent: soluble protein antigens and membrane proteins primarily induce IgG1 but are also accompanied with lower levels of other subclasses, mostly IgG3 and IgG4 [137]. Bacterial capsular polysaccharide antigens induce IgG2 [138], whereas viral infections result in IgG1 and IgG3, with an initial dominant expression of IgG3 [136]. Importantly, IgG3 Abs are particularly effective in the induction of effector functions. IgG4 are also induced upon activation of B cells with allergens or upon helminth or filarial parasite infections [139].

An increasing body of evidence from animal work and human studies suggests a complex role of IgG in atherosclerosis. While several cross-sectional studies showed no correlation between anti-OSE and CVD events [140], a large cross-sectional study of 504 patients demonstrated that anti-oxLDL IgG Abs and IgG Abs against ApoB-100 immune complexes are positively associated with the presence of angiographically determined CVD [141]. Interestingly, Ab levels did not serve as independent predictors of CAD or clinical events [141]. In humans, many cofounding factors and a broader diversity of anti-OSE Abs likely reduce the power of the association between anti-OSE Abs and CVD. Thus, larger studies with strictly defined inclusion/exclusion criteria and a broad spectrum of anti-OSE IgG Ab testing are needed to dissect the implication of IgG in CVD. One of the questions for future studies in humans would also be identification of OSE-specific subclasses of IgGs, characterization of B cell subsets in periphery and within atherosclerotic plaques, and correlation of these parameters with the volume of plaque burden.

In 1995, Palinski and colleagues demonstrate that immunization of *Ldlr*^−/−^ rabbits with MDA-LDL leads to reduced atherosclerosis and increased production of IgM and IgG auto-Abs [142]. Next, it was reported that the titers of anti-oxLDL and anti-MDA-LDL IgG Abs correlate with lesion progression in *Ldlr*^−/−^ mice [143], further suggesting a role for IgG in atherosclerosis. In the next few years, several studies demonstrated that the overall reduction in total IgG upon splenectomy [90] or attenuated anti-oxLDL IgG Ab levels under conditions of bone marrow B-cell deficiency [98] results in accelerated atherosclerotic plaque burden, thus highlighting the overall protective role of anti-OSE Abs. Consequently, more studies have been performed that confirmed the atheroprotective nature of OSE-specific IgGs [144]. Interestingly, human Fab fragment Abs or single-chain variable fragments of anti-OxLDL Abs, which lack immunological properties of intact Abs reduce foam cell formation and atherosclerosis, suggesting that neutralizing functions of anti-oxLDL Abs play an important protective role [145]. Overall, it might be a potential therapeutic value in approaches to increase levels of anti-OSE-specific Abs through passive immunization and/or via active immunization with OSE antigens, but further large size human studies are required to decisively establish a correlation between different isotypes of anti-oxLDL and CVD.

To further understand the role of mature B2 cells and B2-derived IgG Abs, B2 cells were depleted with anti-CD20 Abs in chow diet-fed *Apoe*^−/−^ mice and Western diet-fed *Apoe*^−/−^ or *Ldlr*^−/−^ mice [95,96]. Treatment with anti-CD20 Abs led to the depletion of B2 cells and significant reduction in anti-oxLDL IgG Abs, small decrease in B1 cells and anti-oxLDL IgM, and surprisingly, attenuated atherosclerosis [95,96]. While Kyaw and colleagues found that these effects were independent of the presence of Rag 2-dependent T cells, NKT, and NK cells, suggesting that B2 B cells can directly promote atherogenesis, Ait-Oufell et al. found that B2 cell depletion led to a reduction in DC and Th1 cell activation [95,96]. It would be important to mention that anti-CD20 Abs likely deplete both atheroprotective MZ B cells and atherogenic FO B cells, suggesting that in a combination of MZ and FO B cells, FO B cells likely play a dominate role in atherogenesis. Follow up studies using BAFF receptor-deficient mice further confirmed the overall pathological role of B2 cells [99,146].

While the role of anti-OSE IgM Abs is fairly established, the implication of IgG Abs in inflammatory responses in atherosclerosis is under current active investigation. It is possible that oxLDL-specific IgGs play a dual role in circulation and within the tissues as pro- and anti-atherogenic functions have been reported for IgGs. Anti-oxLDL IgG can neutralize and support clearance of oxLDL via the formation of oxLDL/IgG complexes that are rapidly removed via Fc receptor-dependent uptake in the liver or spleen [147]. Adoptive transfer of human IgG1 Abs against MDA-modified ApoB-100 peptides reduces the extent of atherosclerosis as well as the plaque content of oxLDL epitopes and MΦs in the recipient *Apoe*^−/−^ mice [148]. In line with this study, immunization with ApoB-100 peptide, against which high levels of IgG and IgM Abs are present in healthy human controls, reduces atherosclerosis in *Apoe*^−/−^ mice by about 60% [149]. Altogether, these in vivo data point out the atheroprotective effects of IgGs, and further studies are necessary to characterize the specific mechanisms by which immunization with mLDL and IgG infusion suppress atherogenesis in vivo.

Intriguingly, novel sets of data also support the existence of pathogenic IgG Abs that specifically developed in atherosclerosis-prone conditions. Injection of total IgGs isolated from *Apoe*^−/−^ mice, but not C57BL/6 mice, was sufficient to accelerate atherosclerosis in *Ldlr*^−/−^ mice, supporting the notion that the direct effects of atherosclerosis-derived IgGs are proatherogenic [100]. FcγR are the most important MΦ receptors involved in the clearance of immune complexes containing native or oxLDL via binding the constant region of Abs. FcγR-deficient *Apoe*^−/−^ mice display a decreased plaque formation in comparison with *Apoe*^−/−^ mice, revealing a proatherogenic role of IgG and IgG/immune complexes via MΦ activation [150]. The pathogenic role of IgG was also revealed in several studies devoted to functions of GC B cells and Tfh cells in atherosclerosis. GCs are developed during atherosclerosis, and IgG-producing cells are detected in these structures. Genetic deletion of Tfh cells and therefore, a reduction in GC B cells leads to attenuated atherosclerotic plaque burden in *Apoe*^−/−^ as well as *Ldlr*^−/−^ mice, whereas a promotion of GC formation via dysfunctional CD8+Tregs leads to accelerated atherosclerosis [151], indicating that GC-dependent IgGs play a proatherogenic role [56,152]. While these studies suggest a pathological role of IgG in atherosclerosis, a specific impact of Abs, particularly IgG Abs, remains further to be clarified in comparison with other functions of B cells such as cytokine production or an impact of B cells as antigen-presenting cells.

To delineate the effects of Abs vs. antigen-presenting capacities of B cells and cytokine-associated regulation of the immune response, B cells from *Xbp1^fl^*^/*fl*^*Cd79a^cre^*^/*+*^ (X-box binding protein-1) floxed mice have been used for bone marrow transplantation into *Ldlr*^−/−^ recipients [153]. XBP-1 governs late events in plasma cell differentiation and supports signaling via the BCR but is not required for antigen-specific memory B cell development [154]. In *Xbp1^fl^*^/*fl*^*Cd79a^cre^*^/*+*^*Ldlr*^−/−^ recipients, serum levels of IgG, IgE, and IgM are significantly attenuated, and this Ab deficiency accelerates atherosclerosis. Downregulation of BCL6 and PAX5, responsible for maintaining naive B-cell phenotypes, supports plasma cell differentiation with a subsequent upregulation of plasma cell transcription factors XBP-1, BLIMP-1 (encoded by *Prdm1* gene), and IRF4 [155]. BLIMP-1-deficient B cells from bone marrow *Prdm1^fl^*^/*fl*^*Cd23^cre^*^/*+*^*Ldlr*^−/−^ mice display reduced plasma cell numbers, IgG and IgM levels, and alleviated atherosclerosis, thus further suggesting an atherogenic role of plasma B cells [100]. Additionally, treatment of *Prdm1^fl^*^/*fl*^*Cd23^cre^*^/^*+Ldlr*^−/−^ mice with purified IgG from atherosclerotic, but not healthy, *Ldlr*^−/−^ mice accelerated formation of the necrotic core and plaque burden in recipients [100]. The discrepancy in the results between these two studies might be potentially explained by different effects of BLIMP-1 and XBP-1 on B cell phenotype and functions due to the different effects on plasma cells reflected in the fold of decrease in Ab production in two reports. Additionally, while the overlap of BLIMP-1 and XBP-1 functions is restricted to the unfolded protein response, BLIMP-1 serves at the upstream of XBP-1 and uniquely regulates mTOR activity and plasma cell size [156], and therefore might have additional effects on the B cell population in atherosclerosis. Recently, Centa and colleagues addressed a question on the overall role of immunoglobulins in atherosclerosis using hypercholesterolemic mice that have a defect in Ab production within an otherwise normal immune system using *Prdm1^fl^*^/*fl*^*Cd19^cre^*^/*+*^*Apoe*^−/−^ and control *Prdm1^fl^*^/*+*^*Cd19^cre^*^/*+*^*Apoe*^−/−^ mice [157]. In agreement with Tellier and colleagues, this study demonstrated an overall pathological role of Abs in atherosclerosis via modulation of aortic plaque MΦs. Moreover, this study also demonstrates that IgG Abs are largely derived from the GC activities in the conditions of atherosclerosis. Unexpectedly, the absence of plasma cell-derived IgG and IgM Abs increased subintimal lipid content, VCAM-1 expression, and aortic inflammation in *Prdm1^fl^*^/*fl*^*Cd19^cre^*^/*+*^*Apoe*^−/−^ mice that was accompanied by increased plaque rupture [157] and was dependent on T and B cell interactions. This discovery adds complexity in our understanding of the role of IgG in CVD, and a better insight into mechanisms by which Igs and plasma cells may have a beneficial impact on plaque stability is necessary. Additionally, further investigation of the impact of GC-derived IgGs on plaque stability in humans would be important in order to develop better approaches for the generation of effective vaccines preventing progression of atherosclerosis.

While OSE-derived antigens induce humoral response in atherosclerosis, high-throughput single-cell analysis of the atherosclerosis-associated Ab repertoire in Western diet-fed *Ldlr*^−/−^ mice revealed a more complex landscape of Abs associated with atherosclerosis, as 56 Abs expressed by in vivo-expanded clones of B cells were identified in the analyzed mice [158]. Moreover, a significant proportion of the expanded Abs have been reactive to atherosclerotic plaques, indicating that these Abs are developed against plaque antigens. ALDH4A1, a mitochondrial dehydrogenase involved in proline metabolism, was identified as a target antigen of one of these autoantibodies [158]. Mechanistically, infusion of A12 Abs against ALDH4A1 diminished atherosclerosis progression, highlighting an atheroprotective role of these Abs. With the focus on GC B cells, Lorenzo and colleagues further established that Western Diet-fed *Ldlr*^−/−^ mice have a skewed isotype distribution of class-switched Abs, with a higher proportion of IgG2 subtypes and a lower proportion of IgG3 subtypes and a higher somatic hypermutation and IgG2 bias [158]. Further studies that will investigate how anti-ALDH4A1 Abs protect against atherogenesis are necessary.

## 6. Antigen Presentation and Co-Stimulatory Molecules

B cells detect and bind antigens with their specific BCR process antigens intracellularly and present them as peptide fragments on MHC-II molecules to CD4^+^ cells [159]. Co-stimulatory molecules such as CD40, CD80, CD86, and ICOS support this T cell–B cell interaction. It is also important for B cells to receive the necessary stimuli for GC formation, Ab affinity maturation, and the development of B cell memory, enabling B cells to efficiently respond to invading pathogens and take part in immune responses. Furthermore, MHC-II-dependent interactions between B and T cells provide important signals for T cells to exert their respective effector functions.

Global MHC-II deficiency aggravates atherosclerotic lesions in *Ldlr*^−/−^ mice due to a decrease in Tregs, despite a decrease in systemic inflammation [160]. In this model, the role of B cells is difficult to estimate, as MHC-II-mediated antigen presentation is also performed by DCs and to a lesser extent, by MΦs. The role of MHC-II specifically on B cells is less clear and the type of antigen presented to T cells remains an open, heavily discussed question in the field. There are several conflicting papers on the B cell-specific role of MHC-II in atherogenesis, which mainly vary in their approach to induce B cell-specificity and in the study outcome. In the study from Kyaw and colleagues, B cell-deficient (*µMT*^−/−^*Apoe*^−/−^ mice) were adoptively transferred with either WT or MHC-II-deficient splenic B cells. Transfer of WT B cells led to a drastic increase in atherosclerosis due to the infiltration of activated CD4 T cells into atherosclerotic lesions; however, this effect was significantly reduced when MHC-II-deficient B cells were used for adoptive transfer [161]. In the study from Williams and colleagues, CD19 cre recombinase was used to either delete or overexpress MHC-II specifically in B cells from *Ldlr*^−/−^ mice [162]. No difference in atherosclerotic lesion size nor in lesion immune cell content was observed. Considering these two opposing results, one may speculate the presence of additional factor(s) necessary for B cell- T cell interactions that could be differentially regulated in both mouse models. One of these factors could be differences in lymphoid structures, which are known to be dependent on B cells. Moreover, with the discovery of ATLOs, a local territory for T cell–B cell interactions close to the atherosclerotic aorta has been identified [21]. Depletion of these structures by smooth muscle cells-specific knockout of LTβR revealed their protective role in aged *Apoe*^−/−^ mice by regulated T cell homeostasis [16]. Another potential factor could be that MHC-II deletion skews B cell development and B cell subset distribution, although the total number of B cells, MZ B cells, and T-1 and T2- immature B cells are not affected in the spleen of global *MHC-II*^−/−^ mice [163]. Moreover, it is difficult to differentiate between the role of MHC-II-dependent antigen presentation in inflammatory priming of CD4^+^ cell and its role in collaboration with Tfh cells. Presentation of processed peptide antigen to Tfh cells is important for high affinity class switch Ab production. Mixed *Ldlr*^−/−^ chimera (substituted with 80% *µMT*^−/−^ and 20% *MHC-II*^−/−^ bone marrow) showed a significant decrease in atherosclerosis accompanied by reduced IgG plasma levels [100]. This reduction in atherosclerotic lesion size was associated with lower numbers of splenic INFγ+ and TNFα+ CD4+ T cells and could also be observed in *Apoe*^−/−^ mice adoptively transferred with MHC-II-deficient B cells [161]. Overall, a factor that is worth considering is that unlike mice that only bear one MHC-II allele, humans express more than 10,000 different leukocyte antigen allelic forms, which worsens prediction in autoimmune diseases [101].

In comparison to DCs and MΦs, B cells are relatively poor antigen-presenting cells, and it is still a matter of debate if B cells are able to prime naïve T cells in vivo or if they reactivate memory T cells [164]. Some studies indicate that antigen-specific contact between naïve B and T cells takes place in the absence of DCs and induces proliferation of T cells with a regulatory phenotype with continuous L-selectin expression [165]. Other studies from B cell-deficient mice report that T cell priming by B cells is limited [166] or redundant [167]. More studies are necessary to pinpoint the exact effect of B cell-specific antigen presentation in the context of atherosclerosis.

Another big question in the field remains regarding the source of potential antigens detected by B cells in atherosclerosis. Considering that a lipid-rich environment triggers the development of atherosclerosis, many research groups speculate that LDL and especially oxLDL could serve as potential antigens. Early work from immunization of *Ldlr*^−/−^ mice with either MDA-modified or native LDL shows a reduction in atherosclerosis in both models; however, high Ab titers could only be observed in the mice immunized with MDA-LDL [168]. Due to the success of immunization with LDL, vaccination against its main protein component ApoB has gained attention and was shown to be effective in reducing atherosclerosis in several studies [169,170,171]. However, the precise underlying mechanism is still unclear as well as the role of MHC-II-mediated antigen presentation by B cells. Most studies focused on the induction of Tregs [169,172] and CD4^+^ and CD8^+^ T cell responses, although an increase in antigen-specific IgG1 and IgG2c was observed as well [171].

Tregs dampen immune responses by secretion of anti-inflammatory cytokines such as IL-10 and TGF𝛽 as well as removal/sequestration of (co)stimulatory molecules and therefore, have been attributed a beneficial role in atherosclerosis [173]. Tregs are able to modulate B cell responses either directly by B cell killing via perforin/granzyme B [174] or suppression of B cells functions (e.g., isotype switching) by cell–cell contact or indirectly by inhibiting Th and APC responses, e.g., by downregulating ICOS [175,176]. The balance between Th and Treg is important to maintain peripheral B cell anergy and to prevent activation of autoreactive B cells [177,178]. In the bone marrow, Tregs localize in close proximity to plasma cells and therefore, have been attributed a role in the regulation of long-term humoral memory [179]. Moreover, a specialized subset of Tregs, termed T follicular regulatory cells (T_fr_), has been identified close to lymph follicles that suppress Tfh and germinal center responses [180,181]. Recent studies indicate that B cells, in turn, are able to induce Tregs (CD4^+^CD25^+^Foxp3^−^) cells, which have been named Treg-of-B cells [182] and play a protective role in several inflammatory mouse models. It remains to be determined how Tregs and ex-Tregs with attenuated Treg phenotype (Th17/Tregs [183], Tfh/Tregs [152], and Th1/Tregs [184]) influence the activity of different B cell subsets in atherosclerosis.

Binding of antigens to the BCR initiates signaling cascades within the B cell that not only change the physiological state of the B cell, but also initiate collaboration with T cells by the expression of co-stimulatory surface molecules such as CD80, CD86, and CD40. Data from global CD40-deficient mice indicate that CD40 expression is essential for T cell-dependent immunoglobulin class switch and GC formation, but not for T cell-independent IgM and IgG responses [185]. Using adoptive transfer of CD40-deficient B cells into *µMT*^−/−^*Apoe*^−/−^ mice [161] or mixed *Ldlr*^−/−^ chimera (80% *µMT*^−/−^, 20% *Cd40*^−/−^ BM) [100], Tay and colleagues demonstrated that B cell-specific CD40 deficiency diminishes the development of atherosclerosis in the aortic sinus and in the innominate artery. Mechanistically, CD40 deficiency resulted in a ~70% reduction in GC B cells, Tfh cells, and IgG Abs in mice [100]. While CD40 deficiency in mice clearly identified CD40 expression as a supporting proatherogenic molecule, in humans, current data represent a more complex situation. In a large human follow-up study, different roles have been identified for the expression of CD86 and CD40 on peripheral B cells; whereas high levels of CD19+CD40+ B cells were associated with a decreased risk of stroke, the opposite association was found for CD19+CD86+ cells [186]. Therefore, it would be interesting to see if these discrepancies could be explained by the different expression of CD40 on B cell subsets, which vary between human and mice.

Increasing evidence suggests that B cell subsets are also actively involved in the regulation of Tfh cell differentiation in atherosclerosis. Recently, Nus and colleagues uncovered a role of MZ B cell-specific PDL-1 in the shaping of the Tfh cell population in atherosclerosis [56]. Upon hyperlipidemia, MZ B cells upregulated the surface expression of PDL-1 in an ATF3-dependent manner, leading to PDL-1-mediated suppression of Tfh cell motility, alteration of Tfh cell differentiation, and suppression of Tfh-cell-dependent inflammatory responses [56].

Whereas ICOS-ICOSL signaling between CD4 T cells and DCs plays an important role in the polarization of Tfh and is independent of B cells [187], the presence of B cells is required to maintain BCL6 expression and Tfh polarization. *Ldlr*^−/−^ mice transplanted with ICOS-deficient BM showed a ~40% increase in the atherosclerotic burden accompanied with impaired Treg suppressive functions [188]. In line with that, treatment of mice with Abs against ICOSL led to a reduction in atherosclerosis due to a decrease in Tfh and GC B cells [152]. In arthritis, B cell-specific ICOSL deficiency led to a suppression of the Teff cell-produced cytokines INFγ and IL-17 and the number of Tfh cells [189] alike, indicating a B cell-specific ICOSL may play a similar role in the development of atherosclerosis.

The role of CD80 and CD86 expression on B cells in atherosclerosis is less clear. Buono and colleagues reported reduced atherosclerotic lesions in *Ldlr*^−/−^ mice deficient in both CD80 and CD86 due to decreased priming of antigen-specific lesional T cells [190]. In contrast, another study using irradiated BM chimeric mice of CD80/CD86 found a twofold increase in atherosclerosis accompanied by a decrease in Tregs [191]. An additional interaction pathway of B cells to regulate Treg response in atherosclerosis was identified by the B cell-specific overexpression of glucocorticoid-induced tumor necrosis factor family-related protein ligand (GIRT-L) [192]. Chimeric *Ldlr*^−/−^ *GIRTL-Tg* mice exert significantly less atherosclerosis by regulating the balance between regulatory and effector T cells [192]. Overall, it is likely that the expression of co-stimulatory molecules by B cells influences atherosclerotic lesion development by regulation of T cell homeostasis, but further experiments with different approaches are necessary to better understand the impaction of co-stimulatory molecules on atherosclerosis.

## 7. B Cell Cytokines

Besides their role as antigen-presenting and Ig-secreting cells, B cells secrete cytokines, which have the potential to either promote (TNFα, INFγ, IL-12) or ameliorate (IL-2, IL-4, IL-10, TGFβ) the development of atherosclerosis. Three different cytokine profiles have been identified in B cells: secretion of IL-10 and TGFβ by Bregs, release of IFNγ, IL-12, and TNFα from effector 1 (Be1) B cells, and production of IL-2, IL-4, TNFα, and IL-6 by Be2 cells [67]. Most of these cytokines are produced upon antigen contact and upon priming by Th1 or Th2 cells. In addition, IRA B cells secrete GM-CSF. Whereas IRA B cells aggravate the development of atherosclerosis by skewing leukocyte responses towards INF𝛾-producing Th1 cells [92], the role of B cell-specific release of the other cytokines in atherosclerosis is less clear.

Most research has been conducted on the inflammatory cytokine TNFα, which is known for its disease-aggravating role in atherosclerosis. TNFα-expressing B cells have been found in lesions of both humans [193] and mice [100] and their numbers are increasing under conditions of a high fat diet [161]. *Ldlr*^−/−^ mixed chimeras (80% *Tnfa*^−/−^, 20% *µMT*^−/−^) showed a decrease in plague burden by 30%, which was associated with reduced TNFα expression by other cell types in the atherosclerotic plaque such as MΦs, indicating a cell type overlapping autoinduction loop. Moreover, B cell-derived TNFα was identified to exacerbated apoptosis and necrotic core formation in lesions. Interestingly, injection of TNFα-deficient B2 cells failed to increase atherosclerosis in B cell-deficient or lymphocyte-deficient *Rag2*^−/−^ *Apoe*^−/−^ mice, whereas WT B2 aggravated plaque size by 80%. Recently, the existence of a NLRP3 inflammasome in B cells has been reported that can be activated by BAFF engagement to BAFFR and leads to the secretion of IL-1β [194]. Future studies will clarify how NLRP3 activation might affect the development of atherosclerosis.

IL-10 is an anti-inflammatory cytokine known to decrease atherosclerosis when expressed by leukocytes [195]. IL-10^+^-expressing B cells are decreased in human atherosclerosis and negatively correlate with the inflammatory markers in patients with CVD [196]. Several different B cell subsets such as Bregs, B1, and CD1d^high^CD5+ cells are able to produce IL-10 and affect in vitro T cell diffraction and proliferation in a different manner [197]. Most potent among them are B2-derived CD21^hi^CD23^hi^CD24^hi^ B cells that are located within the draining lymph nodes of *Apoe*^−/−^ mice and suppress atherogenesis in an IL-10-dependent manner when adoptively transferred [198]. Moreover, B cell-derived IL-10 was shown to be important in controlling autoimmunity [199] as well as diabetes and adipose tissue inflammation [200]. Intriguingly, no effects on atherogenesis were observed in *Ldlr*^−/−^ mice with B cell-specific deletion of IL-10 [201]. Interestingly, in the presence of angiotensin II, adoptive transfer of B cells into B cell-deficient *Apoe*^−/−^ *Baffr*^−/−^ mice prevented the progression of atherosclerosis and was associated with a significant increase in IL-10 producing regulatory CD1^high^CD5+ B cells [202]. Overall, the role of B cell-derived cytokines in the development of atherosclerosis is only in its infancy and further work is necessary to shed light on how cytokines secreted by B cells affect the complex orchestration of immune responses. It is likely that B cell-derived cytokines play a more subtle role in shaping their local environment to guarantee optimal conditions to exert their effector functions, which depend on their subset and their activation state.

## 8. Conclusions

Substantial progress has been made in our understanding of B cell functions in the development and progression of atherosclerosis. Recent studies that have been focused on the role of B cell subsets have generated new insights and opportunities to reveal the components of B cell responses in CVD. However, the multiple functions of B cells (antibody production, cytokine release, and antigen presentation) and a B cell-unique way for adaption to the hyperlipidemic and inflammatory environment add various layers of complexity in the overall effects of B cells in atherogenesis that have not been fully elucidated yet. We are only beginning to recognize how and by which antigens BCR signaling, B cell activation, and Ab production are regulated in atherosclerosis and how the overall humoral responses are dependent on individual B cell subsets, locations within the body, and stages of the disease. We are still far from a comprehensive understanding of key components of B cell subset-specific BCR-dependent and TLR-dependent signaling cascades and their relative collaborative roles in shaping the adaptive immune response in atherosclerosis. Substantial progress has been made in understanding some of the relationships among diet, microbiota, and immune systems, but much more work has to be done on investigation of these relationships in atherosclerosis. Although it is well accepted that OSEs, which are present on oxidized lipoproteins and apoptotic cells, serve as (self)-antigens in the context of atherosclerosis, we expect the existence of various (self)antigens that trigger B cell-dependent responses. Therefore, the balance between different antigen-specific B cells and their respective immunoglobulins will be critical for their pro- or anti-atherogenic potential beyond and in addition to their subset specificity. Moreover, determining unique mechanisms that regulate the threshold for B cell activation and identifying potential B cell subset-specific targets of interventions harbors a tremendous potential for clinical interventions. Finally, yet importantly, most of the summarized knowledge has been gained in pre-clinical models (mainly mouse studies) and has to be proven in the human situation, which is far more complex and often involves a range of comorbidities.

## Figures and Tables

**Table 1:** Characteristics of B cell subsets.

B Cell Subset	Location	Surface Markers	Functions	Experimental Models
B1a	Serosal cavities Spleen-dependent	B220^low/mid^ IgM^hi^ IgD^lo^ CD93- CD43+ CD23- CD5^+^ [89]	Secrete natural IgM [42,81] T cell-independent	Splenectomy with B1a or B cell transfer [87,90]
B1b	Serosal cavities Spleen-dependent	B220^low/mid^ IgM^hi^ IgD^lo^ CD43+ CD23- CD5- [89]	Secrete natural IgM, T cell-dependent and independent responses Isotype switch to IgA [42,81]	*Rag*^−/−^*Apoe*^−/−^ with B1b cell transfer [88]
Innate Response Activator B cells	Spleen Differentiated from TLR activated B1a b cells	B220+ IgM^hi^ CD23^lo^ CD21^lo^ CD138^hi^ CD43^hi^ VLA4^hi^ [91]	Produce GM-CSF [91]	*Csf2*^−/−^*μMT* mice (mixed chimera on *Ldlr*^−/−^) [92]
Marginal Zone B cells	Marginal zone of secondary lymphoid tissues	B220+ IgM^hi^ IgD^lo^ CD21^hi^ CD43- CD23- CD1^hi^ [45]	Shuttle antigens to follicles Migrate to T cell zones Produce mainly IgM plasma cells Isotype switch to IgA or IgG3 T cell-independent	*CD79^cre^*^/*+*^*Rbpjk^flox^*^/*flox*^ (mixed chimera on *Ldlr*^−/−^) [56]
Follicular B cells	Recirculating Follicles of secondary lymphoid tissues	B220+ IgM^lo^ IgD^hi^ CD43- CD23+ CD21^mid^ [45]	T cell-dependent GC formation Isotype switched Igs	*μ*MT with B2 B cell transfer [22,93] Anti-BAFF Abs [94] Anti-CD20 and anti-BAFFR [95,96,97]

## Data Availability

Not applicable.
